# 

**Published:** 1814-05

**Authors:** 


### MONTHLY CATALOGUE OF MEDICAL BOOKS.

ABERNETHY's Lectures on the Probability and Rationality of Mr. Hunter's Theory of Life. 8vo. 4s. 6d.

Beck on Veterinary Medicine and Therapeutics, &c. &c. 8vo. 10s. 6d.

Goodlad's Essay on the Diseases of the Vessels and Glands of the Absorbent System, being the Substance of Observations which obtained the Prize for 1812, offered by the Royal College of Surgeons in London. 8vo 6s. 6d.

Higgin's Experiments and Observations on the Atomic Theory and Electrical Phenomena. 8vo. 6s.

Horsley's Remarks on the Pilcaithly and Dunbarney Mineral Waters in Perthshire. 12mo.

Pears on the Nature and Treatment of Consumption. 8vo. 4s.

Watts's Medical Dictionary, containing an Explanation of the Terms in Surgery, Medicine, Midwifery, Anatomy, Chemistry, &c. &c. Second edition; 12mo. 7s.

---

### *NOTICES TO CORRESPONDENTS.*

Dr. Walker conceiving that he has been "calumniated in the last Med. and Phys. Journal, inasmuch as Dr. WALKER's [not the *London*] Vaccine Institution has been charged with a furtive conduct," desires us to insert the following statement:

"When the managers of the London Vaccine Institution determined on having a Director and Assistant-Director, after the manner of the National Vaccine Establishment, and elected Dr. Walker the Director, he called on Mr. Lawrence, and proposed to him the humiliation of consenting to become Assistant-Director—a mere nominal office, to which no duties were attached. Mr. Lawrence pleasantly observed, that humiliation was profitable to the mind, and consented.

"Mr. Lawrence afterwards disliking that his name should be continued, informed the Director that he intended noticing in the Journal that he took no part in the direction of the Institution, but promised to show him the notice before its insertion. Considerable genius and defective reminiscence sometimes meet in the same individual. Take a second proof of this observation. He did not show the notice. J. W."

*Bond Court, Walbrook.*

*We are obliged to Mr. Weatherhead, and to several of our friends and correspondents, for communications, which will appear in our next Number.*

